# Physiology and Pathology of the Nervous System

**Published:** 1859-12

**Authors:** 


### Selections.

### PHYSIOLOGY AND PATHOLOGY OF THE NERVOUS SYSTEM.

DR. BROWN-SEQUARD.

The principal points which Dr. Brown-Sequard endeavors to establish in connection with the physiology and pathology of the central nervous system are these:

1st. Excitations of the anterior roots of the spinal nerves may be a cause of pain, because these roots, being motor, produce a *cramp*. The pain due to this cramp is what has been erroneously called *recurring sensibility*. Cramps, and several other kinds of painful spasms (of the uterus during parturition, of the sphincter ani in certain cases, &c.) are painful on account of a galvanic irritation of sensitive nerves accompanying muscular contractions.

2d. Our movements seem to be guided by the peculiar sensations we derive from the galvanic irritation of certain sensitive nerves of muscles, while they contract.

3d. The power of transmitting sensitive impressions exists in many parts which are not able to give pain or any other sensation when they are excited by our usual means of irritation; so it is with the gray matter of the spinal cord and with many parts of nerves, which, however, are conductors of sensitive impressions.

4th. Hyperæsthesia is a constant result of certain injuries or alterations of the posterior parts of the cerebro-spinal axis, from the tubercular quadrigemina down to the lower end of the spinal cord.

5th. The transmission of sensitive impressions, in the spinal cord, takes place chiefly through the gray matter, and partly through the anterior columns; but, before reaching the gray matter, the impressions, in a certain measure, pass through the posterior columns.

6th. The conductors of sensitive impressions from the trunk and limbs decussate in the spinal cord, and not in the encephalon, as was universally admitted.

7th. Although the spinal cord is greatly altered or injured, sensibility, more or less diminished, may persist everywhere, on account of a peculiar arrangement of the conductors of sensitive impressions.

8th. The various kinds of sensitive impressions seem to be conducted by quite distinct nerve-fibres, in the nerves and in the nervous centres, and the place of passage of some of these conductors in the spinal cord seems not to be the same as that of the others, but none of them go up to the sensorium along the posterior columns.

9th. In the upper part of the cervical region of the spinal cord, near the medulla oblongata, most of the conductors of the orders of the will to muscles are in the lateral columns, and in the gray matter between these and the anterior columns.

10th. The voluntary motor conductors decussate at the lower part of the oblong medulla, and not all along the median line of the base of the encephalon.

11th. The posterior columns of the spinal cord have a great share in reflex movements, and this is the principal cause of the peculiar kind of paralysis so often observed in cases of alteration of these columns.

12th. The effects of excitation of the vaso-motor nerves consist essentially in a contraction of blood-vessels, which is followed by a diminution in the quantity of blood, in the temperature, and in the activity of nutrition. The effects of interruption of continuity of the vaso-motor nerves (*i. e.;* their paralysis) consist essentially in a paralytic dilatation of blood-vessels, which is followed by a greater afflux of blood, an increase of temperature, and a greater activity of nutrition.

13th. As a great many vaso-motor nerve-fibres go up to the brain, and to the cerebellum along the spinal cord, the medulla oblongata and the pons Varolii, the diseases or injuries of the various parts of the cerebro-spinal axis, besides symptoms concerning sensibility and movement, present symptoms, depending upon irritation or paralysis of vaso-motor nerves; contraction or relaxation of blood-vessels, diminution of temperature, alterations of nutrition, of secretions, &c.

14th. Besides the influence of the nervous system upon nutrition, absorption, and secretion, through the vaso-motor nerves, there is another, which seems to consist in changes in the elements of the tissues—changes producing various modifications in the quantity of blood attracted, and in the interchange of materials between the blood and the tissues.

15th. The absence of the influence of the nervous system on any part of the body is hardly a cause of other alterations of nutrition than atrophy, while the irritation of the nervous system is a most powerful direct or reflex cause of a great many morbid changes in nutrition, secretion, &c.

16th. The sympathetic normal and morbid changes of nutrition, secretion, &c., are reflex phenomena, the study of which shows how many diseases are produced by a reflex action, and how a rational mode of treatment might be arrived at.

17th. The loss of consciousness in simple vertigo or in complete attacks of epilepsy does not depend upon a disease of the brain, but upon a contraction of the blood-vessels of the cerebral lobes—contraction due to some irritation of the vaso-motor nerves of these vessels, either by some direct cause irritating them in the base of the encephalon or the spinal cord, or by a reflex influence.

18th. Much more frequently than has been imagined, all the following affections may be produced by a peculiar kind of irritation starting from almost any centripetal part of the nervous system: epilepsy, the various forms of insanity, chorea, catalepsy, hysteria, tetanus, hydrophobia, &c.

19th. The medulla oblongata is neither the only nor an essential nervous centre for the respiratory movements.

20th. There are a great many nerve-fibres and nerve-cells in the medulla oblongata, the pons Varolii, and the other parts of the base of the encephalon, which are not employed in the transmission of sensitive impressions or of the orders of the will to muscles, and are endowed with the singular property of producing, after even a slight irritation, a *persistent* spasm in certain muscles, and especially in the neck. Rotatory convulsions very often depend chiefly upon the production of such spasms, and in changes of the blood-vessels of certain parts of the encephalon.

21st. The irritation of the auditory nerve may cause rotatory or simple clonic convulsions.

22d. The conductors of the orders of the will to muscles, of the sensitive impressions, and of the nervous influences to blood-vessels, decussating at different places in the cerebro-spinal axis, various symptoms are to be observed, depending upon either the irritation or the paralysis of these three kinds of conductors, according to the part of a lateral half of the cerebro-spinal axis where an alteration exists.

